# Supplementary material for: Ultraviolet radiation shapes dendritic cell leukaemia transformation in the skin
Source: Nature. 2023 Jun 7;618(7966):834–41. doi: 10.1038/s41586-023-06156-8 (PMC10284703; doi:10.1038/s41586-023-06156-8)
Supplement: Supplementary file 1 — Reporting Summary [file 41586_2023_6156_MOESM1_ESM.pdf]

nature research

Corresponding author(s): Lane

Last updated by author(s): Mar 31, 2023

Reporting Summary

Nature Research wishes to improve the reproducibility of the work that we publish. This form provides structure for consistency and transparency in reporting. For further information on Nature Research policies, see our Editorial Policies and the Editorial Policy Checklist.

Statistics

For all statistical analyses, confirm that the following items are present in the figure legend, table legend, main text, or Methods section.

n/a	Confirmed	
☐	☒	The exact sample size (*n*) for each experimental group/condition, given as a discrete number and unit of measurement
☐	☒	A statement on whether measurements were taken from distinct samples or whether the same sample was measured repeatedly
☐	☒	The statistical test(s) used AND whether they are one- or two-sided<br>*Only common tests should be described solely by name; describe more complex techniques in the Methods section.*
☐	☒	A description of all covariates tested
☐	☒	A description of any assumptions or corrections, such as tests of normality and adjustment for multiple comparisons
☐	☒	A full description of the statistical parameters including central tendency (e.g. means) or other basic estimates (e.g. regression coefficient) AND variation (e.g. standard deviation) or associated estimates of uncertainty (e.g. confidence intervals)
☐	☒	For null hypothesis testing, the test statistic (e.g. *F*, *t*, *r*) with confidence intervals, effect sizes, degrees of freedom and *P* value noted<br>*Give P values as exact values whenever suitable.*
☒	☐	For Bayesian analysis, information on the choice of priors and Markov chain Monte Carlo settings
☒	☐	For hierarchical and complex designs, identification of the appropriate level for tests and full reporting of outcomes
☐	☒	Estimates of effect sizes (e.g. Cohen's *d*, Pearson's *r*), indicating how they were calculated

Our web collection on statistics for biologists contains articles on many of the points above.

Software and code

Policy information about availability of computer code

Data collection	No software was used
Data analysis	All software and code are listed in the Methods section, including: BWA version 0.7.15; Picard version 2.5.0; GATK version version 4.0.0.0; Mutect2; MutationalPatterns; STAR version 2.6.0c; cellranger version 7.0.0; bcftools version 1.10.2; samtools mpileup; data.table; IronThrone-GoT version 2.1; Seurat version 4.1.1; Harmony; randomForest version 4.7-1.1; GeneSetEnrichmentAnalysis Molecular Signatures Database version 7.1; Custom R Scripts written for the analysis of scRNA-seq data will be on Github (https://github.com/petervangalen/Single-cell_BPDCN/) and and as DOI: 10.5281/zenodo.7746255 (https://zenodo.org/record/7746255#.ZBSOti-B0eY).

For manuscripts utilizing custom algorithms or software that are central to the research but not yet described in published literature, software must be made available to editors and reviewers. We strongly encourage code deposition in a community repository (e.g. GitHub). See the Nature Research guidelines for submitting code & software for further information.

Data

Policy information about availability of data

All manuscripts must include a data availability statement. This statement should provide the following information, where applicable:

- Accession codes, unique identifiers, or web links for publicly available datasets
- A list of figures that have associated raw data
- A description of any restrictions on data availability

The WES/WGS data are available in dbGaP (https://dbgap.ncbi.nlm.nih.gov/), accession number phs003228.v1.p1. For single-cell analyses, sequencing data and gene expression matrices are available in the Gene Expression Omnibus (https://www.ncbi.nlm.nih.gov/geo/), accession number GSE227690. Human genome reference hg19 is available at https://www.ncbi.nlm.nih.gov/data-hub/genome/GCF_000001405.13/.

Field-specific reporting

Please select the one below that is the best fit for your research. If you are not sure, read the appropriate sections before making your selection.

☒ Life sciences ☐ Behavioural & social sciences ☐ Ecological, evolutionary & environmental sciences

For a reference copy of the document with all sections, see nature.com/documents/nr-reporting-summary-flat.pdf

Life sciences study design

All studies must disclose on these points even when the disclosure is negative.

Sample size	Sample size was based on tissue availability in this rare cancer.
Data exclusions	No data were excluded.
Replication	DNA mutations were confirmed by orthogonal methods on the same samples: e.g., exome capture and targeted capture/PCR amplification-based sequencing. Laboratory data were generated in at least two biologically independent experiments, each with at least three replicates.
Randomization	Randomization not relevant to this study as all data are sequencing-based approaches to human cancer tissue samples. Participants were known to have a diagnosis of this rare leukemia and consented to have their tumor tissue collected and sequenced.
Blinding	No blinding was performed. Sequencing was on samples from patients with known diagnoses of BPDCN or from normal healthy donors whose status was known to the research team. In laboratory experiments, the researchers were required to generate experimental models and conditions and were therefore aware of the status of each. Pathologists who evaluated the tissue samples were blinded to whether the patient had known skin and/or bone marrow involvement with BPDCN prior to seeing the tissue sections.

Reporting for specific materials, systems and methods

We require information from authors about some types of materials, experimental systems and methods used in many studies. Here, indicate whether each material, system or method listed is relevant to your study. If you are not sure if a list item applies to your research, read the appropriate section before selecting a response.

Materials & experimental systems

n/a	Involved in the study	
☐	☒	Antibodies
☒	☐	Eukaryotic cell lines
☒	☐	Palaeontology and archaeology
☐	☒	Animals and other organisms
☐	☒	Human research participants
☒	☐	Clinical data
☒	☐	Dual use research of concern

Methods

n/a	Involved in the study	
☒	☐	ChIP-seq
☒	☐	Flow cytometry
☒	☐	MRI-based neuroimaging

Antibodies

Antibodies used	CD11b Alexa Fluor 700 BioLegend 101222 M1/70 B336447;<br>CD11c PE/Cyanine7 BioLegend 117317 N418 B346714;<br>B220 APC/Cyanine7 BioLegend 103224 RA3-6B2 B321245;<br>Siglec-H PE BioLegend 129605 551 B248184;<br>MHC Class II PerCP/Cyanine5.5 BioLegend 107626 M5/114.15.2 B269461
Validation	Examples per manufacturer's websites of flow cytometry in mouse cells.

Animals and other organisms

Policy information about studies involving animals; ARRIVE guidelines recommended for reporting animal research

Laboratory animals	wild-type C57BL/6 mice, male consistent with extreme male bias of BPDCN
Wild animals	*Provide details on animals observed in or captured in the field; report species, sex and age where possible. Describe how animals were caught and transported and what happened to captive animals after the study (if killed, explain why and describe method; if released, say where and when) OR state that the study did not involve wild animals.*

Field-collected samples	*For laboratory work with field-collected samples, describe all relevant parameters such as housing, maintenance, temperature, photoperiod and end-of-experiment protocol OR state that the study did not involve samples collected from the field.*
Ethics oversight	*Identify the organization(s) that approved or provided guidance on the study protocol, OR state that no ethical approval or guidance was required and explain why not.*

Note that full information on the approval of the study protocol must also be provided in the manuscript.

Human research participants

Policy information about studies involving human research participants

Population characteristics	Population is patients age 18 and over with BPDCN who presented to the Dana-Farber Cancer Institute for treatment and consented to tissue banking for research. All patients in this population were offered the choice to consent to a sample banking protocol if they chose. Healthy controls were volunteer donors consented to tissue banking protocols at Brigham and Women's Hospital.
Recruitment	Participants were not recruited. All patients with blood cancers at Dana-Farber Cancer Institute are offered participation in an excess tissue sample banking protocol for de-identified research. The research team is not aware of any additional self-selection biases that may have influenced the population, and it is generally in keeping with the total population of patients with BPDCN. Normal healthy donors were volunteer bone marrow donors age 18 and over who elected to participate in marrow donor registries without compensation. There are no known biases in the selection of the normal donor population.
Ethics oversight	Dana-Farber Cancer Institute and Brigham and Women's Hospital Institutional Review Boards (IRB) approved all studies.

Note that full information on the approval of the study protocol must also be provided in the manuscript.
